# Nonfatal diseases and quality of life: perspectives in Brazil

**DOI:** 10.1590/1516-3180.2020.1381311219

**Published:** 2020-02-27

**Authors:** Isabela Martins Benseñor, Paulo Andrade Lotufo

**Affiliations:** I MD, PhD. Full Professor, Department of Internal Medicine, Faculdade de Medicina FMUSP, Universidade de Sao Paulo, São Paulo (SP), Brazil.; II MD, DrPH. Full Professor, Department of Internal Medicine, Faculdade de Medicina FMUSP, Universidade de Sao Paulo, São Paulo (SP), Brazil.

Some diseases kill many people, such as cardiovascular diseases, the most frequent cause of death worldwide. Other diseases do not kill, and they are classified as nonfatal. However, they can compromise the quality of life in such a way that life becomes a huge burden. This burden is quantified in terms of the years lived with disability (YLD). For fatal diseases, the burden is quantified as the YLD plus the years of life lost (YLL). The sum of YLD plus YLL is called disability-adjusted life years (DALYs).

In 1990, coronary heart diseases were the second greatest cause of DALYs. They reached the first position in 2005 and have maintained this position ever since. In 1990, low-back and neck pain were in the seventh position as causes of DALYs, and gradually rose in rank to reach the fourth position in 2013. Depression also rose in rank from the 15^th^ cause of DALYs in 1990 to the 11^th^ position in 2013. In 2013, migraine appeared for the first time as a cause of DALYs, in 25^th^ position.^1^

Considering only YLD, low-back pain occupied the first position in 1990, followed by migraine (2^nd^), depression (4^th^), anxiety (6^th^), other musculoskeletal diseases (7^th^) and neck pain (8^th^). Although some changes in position occurred subsequently, all of these were still among the top ten causes of YLD in 2016, as follows:^2^ low-back pain (1^st^), migraine (2^nd^), depression (5^th^), other musculoskeletal disorders (6^th^), neck pain (7^th^) and anxiety (9^th^). In the specific case of Brazil in 2016, low-back pain occupied the first position, followed by migraine (2^nd^), anxiety (3^rd^), depression (4^th^), other musculoskeletal disorders (5^th^) and neck pain (7^th^).^2^

Data from 2017 described the pattern of conditions associated with DALYs according to sex. In 2017 among women, low-back pain occupied the first position, followed by headaches (2^nd^), depression (3^rd^), anxiety (8^th^), neck pain (9^th^) and other musculoskeletal conditions (11^th^); while among men, the ranking was low-back pain (1^st^), headaches (2^nd^), depression (5^th^), other musculoskeletal diseases (10^th^), neck pain (11^th^) and anxiety (13^th^)^3^ Thus, despite some changes in the positions of these conditions according to sex, there was no significant difference in relation to low-back pain, headaches and depression. Moreover, although some variables have been measured differently from one study to another, such as migraines in 2013 and 2016, and headaches in 2017, there has been a clear pattern of association between chronic pain and psychiatric disorders over the years in all studies.

What do these data show us? The information that chronic pain is associated with psychiatric disorders is not new. However, how healthcare services are dealing with the association of chronic pain and psychiatric disorders has reached a critical point. It has been reported that in the United States, an epidemic of misuse of opioids is causing 33,000 deaths per year, through both prescribed and illegal use of opioids.^4^^,^^5^

In a recent review, Stoicea et al. concluded that with approximately 100 million people suffering from both chronic and acute pain in the United States in 2016, opioids would continue to remain highly prescribed medicines across the US.^5^ More than two thirds of overdose episodes in 2016 were opioid-related.^5^

Nonetheless, this is not the first crisis relating to use of opioids over the course of the history of humanity. The ancient Sumerians used a substance medicinally and recreationally around 3300 BC that is thought to have been opium. From there, its use spread across the Middle East and onwards to Greece, India and China. In the 17^th^ century, Portuguese sea merchants profited from exporting opium to China. The British later took over the trade. When the Chinese government tried to block imports, Britain launched two wars to maintain and expand its massively lucrative drug trafficking. As written elsewhere,^6^ “Opium also became used in England and the United States in the form of patent medicines and drinks, which workers consumed to ease the miseries of poverty and parents used to quieten their children”. This usage was correlated with the deaths of several children under the age of five years due to narcotic poisoning between 1863 and 1867.^7^

We are not saying that Brazil is at risk of an opioid epidemic. There are many differences in the structure of healthcare services between Brazil and the United States. Brazil has a National Health System with universal access to healthcare, whereas the United States only has Medicare, Medicaid and, more recently, Obamacare. Furthermore, some pain killers that are available in Brazil, such as dipyrone, are prohibited in the United States. Dipyrone is known as “Mexican aspirin” in the United States.

However, it is now important to discuss combined strategies for dealing with chronic pain and psychiatric disorders in Brazil. A trend involving an association between chronic pain and psychiatric disorders has appeared worldwide, and it has been correlated with high YLD. At the end of the second decade of the 21^st^ century, it is more than time to discuss strategies to improve the quality of life of the Brazilian population. One point is obvious: chronic pain and psychiatric disorders walk together.

In the case of headaches, some studies have suggested that these two disorders have risk factors in common.^8^^,^^9^^,^^10^ The approaches that may be useful in addressing the combination of these two disorders may include introduction of prophylaxis for chronic headaches, treatment of psychiatric disorders and prevention of abuse of pain medication, which is a prevalent cause of headaches.^11^ For low-back pain, it may be beneficial to treat pain symptoms through both pharmacological approaches and alternative therapies such as tai chi or acupuncture.^12^ Some studies have shown that use of tricyclics has a mild effect regarding improvement of low-back pain.^13^ The increasing incidence of obesity and increasing life expectancy in Brazil may complicate the scenario over the coming years through concomitant increases in the incidence of other forms of musculoskeletal pain.

Although some differences according to sex have been observed, the profile of the association of chronic pain and psychiatric disorders is very similar between men and women. This shows that the strategy for addressing the problem can be the same for both sexes.

It is now time to create a strategy for dealing with the association of chronic pain and psychiatric disorders in Brazil, through focusing on diminishing YLD and improving quality of life. Several strategies are possible, beginning with inclusion of pain treatment approaches as part of medical school undergraduate curricula and medical residence training. Most importantly, a solution that is appropriate for Brazil needs to be sought. This should take the structure of the Brazilian healthcare system into consideration and, especially, should be centered on primary care.
